# Evaluation of Reference Genes for Quantitative Real-Time PCR Analysis in the Bean Bug, *Riptortus pedestris* (Hemiptera: Alydidae)

**DOI:** 10.3390/insects14120960

**Published:** 2023-12-18

**Authors:** Liuyang Wang, Qingyu Liu, Pei Guo, Zhanlin Gao, Dan Chen, Tao Zhang, Jun Ning

**Affiliations:** 1 State Key Laboratory for Biology of Plant Diseases and Insect Pests, Institute of Plant Protection, Chinese Academy of Agricultural Sciences, Beijing 100193, China; wliuyang1008@163.com (L.W.); lqy19950716@163.com (Q.L.); 2 Institute of Plant Protection, Hebei Academy of Agriculture and Forestry Sciences, Integrated Pest Management Center of Hebei Province, Key Laboratory of IPM on Crops in Northern Region of North China, Ministry of Agriculture, Baoding 071000, China; peig2021@163.com (P.G.); gaozhanlin@sina.com (Z.G.); feier5915696@163.com (D.C.)

**Keywords:** *Riptortus pedestris*, qRT-PCR, reference genes, expression stability, normalization

## Abstract

**Simple Summary:**

Reference genes serve as a foundation for investigating the functionality of target genes in insects. Nevertheless, the selection of reference genes is highly dependent upon particular experimental circumstances. Recently, the bean bug, *Riptortus pedestris* (Hemiptera: Alydidae), has attracted increasing attention due to its damage on yield losses of soybean crops. However, there is a dearth of literature regarding the evaluation of reference genes for quantitative real-time PCR (qRT-PCR) in *R. pedestris*. The current study aimed to assess the stability of expression levels in 16 candidate reference genes in *R. pedestris* across six experimental conditions. This evaluation was conducted using ΔCt method, GeNorm, NormFinder, BestKeeper, and RefFinder. Our results indicate that the optimum reference genes were as follows: *RPL7A* and *EF1* for the developmental stage, *α−tubulin* and *EF1* for adult tissues, *RPL32* and *RPL7A* for adult age, *RPL32* and *SOD* for nymph age, *RPS23* and *RPL7A* for temperature, and *HSP70* and *GAPDH* for mating status. These findings will provide a basis for future investigations into the expression and function of target genes in bean bugs.

**Abstract:**

Quantitative real-time PCR (qRT-PCR) is widely accepted as a precise and convenient method for quantitatively analyzing the expression of functional genes. The data normalization strongly depends upon stable reference genes. The bean bug, *Riptortus pedestris* (Hemiptera: Alydidae), is a significant pest of leguminous crops and broadly distributed across Southeast Asia. In this study, a total of 16 candidate reference genes (*RPL32*, *RPS23*, *SDHA*, *UBQ*, *UCCR*, *GST*, *TATA*−*box*, *HSP70*, *GAPDH*, *RPL7A*, *SOD*, *RPS3*, *Actin*, α−*tubulin*, *AK*, and *EF1*) were carefully chosen in *R. pedestris*, and their expression levels were assessed across various conditions, including different developmental stages, diverse tissues, temperature treatments, adult age, molting time, and mating status. Following this, the stability of these reference genes was evaluated using four algorithms (ΔCt, GeNorm, NormFinder, and BestKeeper). Ultimately, the comprehensive rankings were determined using the online tool RefFinder. Our results demonstrate that the reference gene for qRT-PCR analysis in *R. pedestris* is contingent upon the specific experimental conditions. *RPL7A* and *EF1* are optimal reference genes for developmental stages. Furthermore, α−*tubulin* and *EF1* exhibit the most stable expression across various adult tissues. *RPL32* and *RPL7A* exhibit the most stable expression for adult age. For nymph age, *RPL32* and *SOD* display the most stable expression. For temperature conditions, *RPS23* and *RPL7A* were identified as the most suitable for monitoring gene expression. Lastly, we verified the practicability of evaluating expression levels of odorant-binding protein 37 (*RpedOBP37*) and cytochrome P450 6a2 (*RpedCYP6*) throughout developmental stages, tissues, and temperature conditions. These findings are a significant addition to the qRT-PCR analysis studies on *R. pedestris*, serving as a fundamental groundwork for future investigations on stable reference genes in *R. pedestris* as well as other organisms.

## 1. Introduction

Quantitative real-time PCR (qRT-PCR) is a highly valuable molecular technique for measuring and evaluating gene expression due to its high sensitivity, specificity, reproducibility, reliability, and capacity for high-throughput analysis [1,2]. The process of data normalization in qRT-PCR assays, to some extent, is influenced by variations in the amount of starting material, the quality and integrity of template RNA samples, the efficiency of reverse transcription, the recovery and integrity of mRNA, as well as the design of primers and transcription efficiency [3,4]. These factors influence the variation of the data and the data analysis; therefore, the normalization method should be chosen to account for all of these variables. Suitable reference genes are commonly introduced to normalize and standardize the experimental samples. These reference genes should remain unaffected by external factors and exhibit stable expression across diverse tissues or cells [5]. In addition, the stability of reference genes alters in response to different experimental circumstances [6]. Thus, it is necessary to ascertain and assess the stability of reference genes across diverse experimental conditions to attain precise and dependable experimental outcomes.

A suitable reference should exhibit consistent and robust expression across all samples, and not co-regulate with the target gene [7]. Historically, reference genes have been considered as housekeeping genes that are presumed to maintain stable and constitutive expression regardless of the physiological conditions in various samples or treatments being investigated [8,9]. In the past decade, many traditional reference genes have been widely utilized as standard markers to evaluate the expression patterns of functional genes, including alpha−tubulin (*α*−*tubulin*), glutathione S-transferase (*GST*), succinate dehydrogenase complex subunit A (*SDHA*), heat shock protein (*HSP20*, *HSP40*, *HSP70*, and *HSP90*), arginine kinase (*AK*), elongation factor 1 (*EF1*), *Actin*, and glyceraldehyde-3-phosphate dehydrogenase (*GAPDH*) [9,10,11,12,13,14]. These reference genes exhibit a high degree of conservation and are implicated in diverse cellular processes, encompassing the cytoskeleton, energy metabolism, protein synthesis, cell differentiation, and so on [10,11,15].

The bean bug, *Riptortus pedestris* Fabricius (Hemiptera: Alydidae), is widely distributed in Asia and other regions, encompassing China, Japan, India, South Korea, Sri Lanka, Myanmar, and Malaysia [16,17,18]. Both nymphs and adults of *R. pedestris* acquire nutrients and water through the insertion of their sucking mouthparts from leaves, stems, pods, and seeds of plants. This feeding behavior leads to significant reductions in crop yields and seed quality [16,19]. Several studies have indicated that *R. pedestris* is a significant contributor to the occurrence of soybean “Staygreen Syndrome” in the Huang-Huai-Hai region of China [20,21,22]. In recent times, the advancement of next-generation sequencing technologies and transcriptome analysis has yielded a substantial volume of genetic data. For instance, Huang et al. reported a considerable number of genes that possess the ability to induce cell death, reactive oxygen species (ROS) burst, and hormone signal alterations in *R. pedestris* [19]. Fu et al. identified numerous differentially expressed genes implicated in wing formation through a comparative analysis of the transcriptomes of *R. pedestris* eggs, first-, second-, third-, fourth-, and fifth-instar nymphs, as well as adults [23]. Moreover, Liu et al. [24] and Li et al. [25] conducted a transcriptional analysis of gene expression in the antennae of *R. pedestris* and identified several genes potentially involved in olfactory and taste transduction. The quantitative assessment of gene expression not only enhances the comprehension of the molecular mechanisms associated with insect development, stress response, and behavioral regulation in *R. pedestris* but also holds the potential to reveal novel targets for effective pest control.

*Actin*, *EF1*, and *GAPDH* have been employed as reference genes to normalize the expression of target genes implicated in the development, odor perception, odor discrimination, and behaviors (such as feeding and mating) of *R. pedestris* [23,24,25,26]. Nevertheless, the aforementioned studies did not assess the appropriateness of these genes in experimental conditions. To screen optimal reference genes for gene expression analyses in *R. pedestris*, we evaluated 16 reference genes which are commonly utilized in insect qRT-PCR studies, including ribosomal protein L32 (*RPL32*), ribosomal protein S23 (*RPS23*), *SDHA*, ubiquitin-conjugating protein (*UBQ*), ubiquinol-cytochrome-c reductase (*UCCR*), *GST*, TATA−box binding protein (*TATA*−*box*), *Hsp70*, *GAPDH*, ribosomal protein L7A (*RPL7A*), superoxide dismutase (*SOD*), ribosomal protein S3 (*RPS3*), *AK*, *Actin*, *EF1*, and α−*tubulin*. We also assessed the impact of developmental stages, tissues, adult age, nymph age, temperature, and mating status on reference gene expression. Our results will contribute to the development of a more reliable approach to normalizing *R. pedestris* qRT-PCR data.

## 2. Materials and Methods

### 2.1. Insect Rearing and RNA Extraction

The bean bug cultures were reared in an insectary of 24 ± 2 °C, 60 ± 10% humidity, and 14:10 L:D photoperiod. Both nymphs and adults were fed with fresh green bean pods (*Phaseolus vulgaris* L.). Total RNA was extracted using TRIzol (TransGen Biotech, Beijing, China). First-strand cDNA was synthesized from 1 mg of total RNA using the All-in-One Super Mix for qPCR Reagent Kit (TransGen Biotech, Beijing, China) according to the manufacturer’s recommendations.

### 2.2. Candidate Reference Genes Selection and Primer Design

The sequences of 16 candidate reference genes (*RPL32*, *RPS23*, *SDHA*, *UBQ*, *UCCR*, *GST*, *TATA*−*box*, *HSP70*, *GAPDH*, *RPL7A*, *SOD*, *RPS3*, *Actin*, α−*tubulin*, *AK* and *EF1*), which have been frequently employed in Hemiptera insects, were obtained from the genome data of *R. pedestris* (GenBank accessions: GCA_019009955.1). Primers for candidate reference genes were designed using Primer 6 software and subsequently synthesized by Sangon Biotechnology Co., Ltd. (Shanghai, China). The purified PCR products were used as the initial template for constructing the standard curve, which allowed for the determination of primer amplification efficiency. Each gradient was diluted by a factor of 2, resulting in a total of five gradients.

### 2.3. qRT-PCR Analysis

qRT-PCR tests were performed on a QuantStudio 3 Real-Time PCR System (Thermo Fisher Scientific Inc., Waltham, MA, USA). The qRT-PCR was conducted by using 1 μL of the cDNA template and 10 μL 2 × TransStart Tip Green qPCR SuperMix (TransGen Biotech, Beijing, China), with 0.5 μL forward and reverse primers (10 μM) and 8 μL ddH_2_O. The thermal cycling conditions consisted of an initial cycle at 94 °C for 30 s, followed by 40 cycles of 5 s at 94 °C, 15 s at 55 °C, and 10 s at 72 °C. Subsequently, a melting curve analysis ranging from 60 °C to 95 °C was performed to ensure the amplified product’s consistency and specificity. For each reaction, three biological replicates and three technical replicates were established.

### 2.4. Determination of Reference Gene Expression Stability

The stability of 16 candidate reference genes was assessed using the ΔCt method [27], as well as the GeNorm [2,28], NormFinder [28], and BestKeeper [5] software programs. The BestKeeper and ΔCt method employ raw quantification cycle (Cq) values, whereas NormFinder and GeNorm utilize expression values calculated as 2^(−ΔCq)^. Ultimately, the online tool RefFinder (https://www.heartcure.com.au/reffinder/, accessed on 8 October 2023) was employed to determine the weighted geometric means in the form of stability values derived from the aforementioned algorithms. This allowed for the establishment of a consensus ranking regarding the stability of the reference genes [29,30,31]. Moreover, the GeNorm algorithm was used to determine the most suitable quantity of reference genes by evaluating pairwise variance values (V). If the ratio (V_n_/V_n+1_) is below 0.15, the optimal number of reference genes is n [2].

### 2.5. Experimental Treatments and Sample Collection

#### 2.5.1. Developmental Stages

In this study, *R. pedestris* samples of various developmental stages were collected, including eggs (100), the first instar nymph (10 individuals), the second instar nymph (five individuals), the third instar nymph (three individuals), the fourth instar nymph (one individual), the fifth instar nymph (one individual), and 2-day-old adults (one male and one female). There were a total of 21 samples comprising seven development stages with three biological replicates each.

#### 2.5.2. Adult Tissues

In a pre-cooled PBS solution, 3-day-old adults were dissected to collect various tissues, including heads (10 individuals), thoraxes (two individuals), abdomens (two individuals), legs (20 individuals), wings (20 individuals), female antennae (30 pairs), and male antennae (30 pairs).

#### 2.5.3. Adult Age

Adults of different age were collected on the 1st, 3rd, 6th, 10th, 15th, and 20th day post-emergence and subsequently preserved at a temperature of −80 °C until RNA extraction. There were a total of 18 samples comprising six treatments with three biological replicates each.

#### 2.5.4. Nymph Age

We collected 5-instar nymphs of post-molt 3, 6, 9, and 12 h, treated in liquid nitrogen and stored at −80 °C until further utilization. There were a total of 15 samples comprising five treatments with three biological replicates each.

#### 2.5.5. Temperature

The 3-day-old females were maintained at temperatures of 15, 18, 20, 25, 28, and 32 °C for two hours. After that, the specimens were quick-frozen in liquid nitrogen and stored at −80 °C. There were a total of 18 samples comprising six temperature treatments with three biological replicates each.

#### 2.5.6. Mating Status

In a plastic vial measuring 4 cm in diameter and 12 cm in height, paired adults were housed until they mated. Meanwhile, individual females or males housed in the vial at the same time were used as unmated samples. Subsequently, we collected mated adults (one male and one female) and unmated adults (one male and one female) for evaluating the influence of mating status, respectively. There were a total of 12 samples comprising mated and unmated adults with three biological replicates each.

### 2.6. Validation of the Candidate Reference Genes

We selected odorant-binding protein 37 (*RpedOBP37*) [25] and cytochrome P450 6a2 (*RpedCYP6*) [26] for the validation of the most stable reference genes (*EF1* and *Actin)* and a variable one (*SDHA*). The forward primer and reverse sequences for *RpedOBP37* were 5′-ATGGGAGCGATTTCTGAT-3′ and 5′-ATAGCGGTTTCACATCCA-3′, while the primer pairs for *RpedCYP6* were 5′-TACGGTCGAGGTTATCTG-3′ and 5′-GGGCTGGTTATCCTTACT-3′. The study examined the variations in the expression levels of the target genes across different developmental stages, adult tissues, and temperature treatments. The 2^−ΔΔC^_T_ method was employed to analyze the relative mRNA expression data of target genes [32].

## 3. Results

### 3.1. Amplification Performance of Primers

Prior to assessing the appropriateness of the reference genes, it is imperative to validate the specificity and efficiency of PCR amplification. Each PCR product subjected to detection using a 1.2% agarose gel presented as a solitary band of the anticipated size. Furthermore, the melting curves of each primer pair’s PCR amplification exhibited a single peak (Figure S1). The amplification efficiency of each primer pair ranged from 95.8% to 115.8%, while all regression coefficients surpassed the threshold of 0.990 (Table 1, Figure S2). Consequently, the primers were suitable for quantitative determination.

### 3.2. Expression Profiles of Candidate Reference Genes

**Figure 1:**
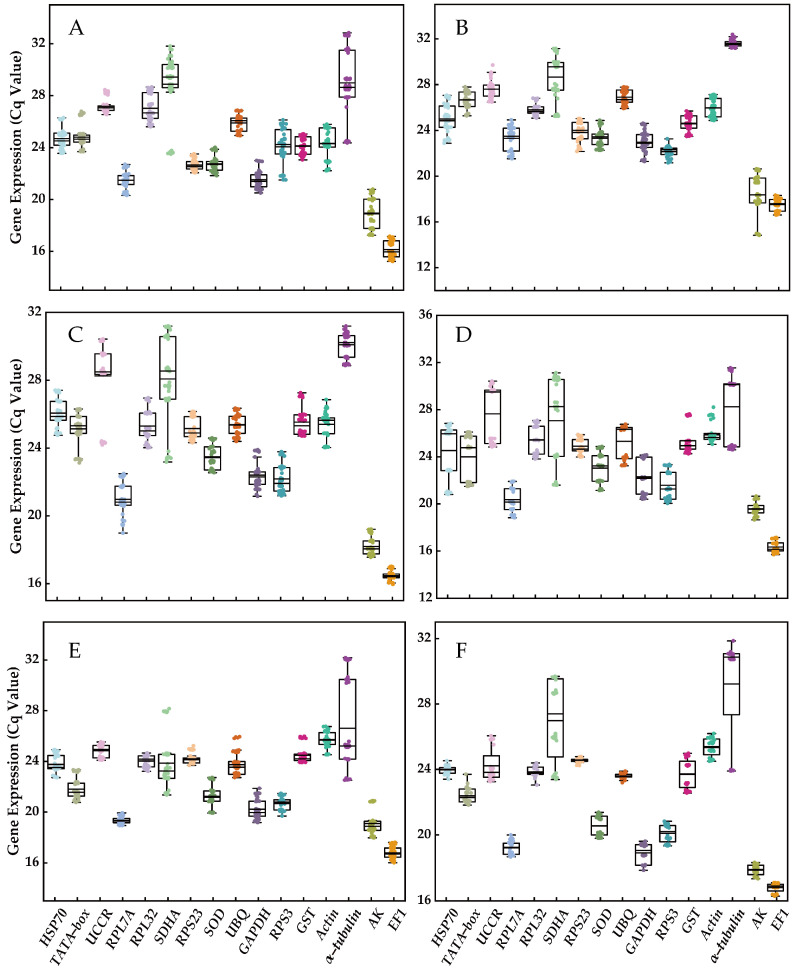
The expression profiles of the 16 candidate reference genes under various experimental conditions. (**A**) Developmental stage, (**B**) different tissue, (**C**) adult age, (**D**) nymph age, (**E**) temperature, and (**F**) mating status. The expression levels of candidate reference genes were quantified using Cq values. The box plot displays the distribution of Cq values, with the box representing the interquartile range (25th to 75th percentiles), the line within the box indicating the median, and the whiskers representing the minimum and maximum Cq values.

*EF1* was identified as the most highly expressed reference gene, followed by *AK*, *RPL7A*, *GAPDH*, *RPS3*, *SOD*, *RPS23*, *TATA*−*box*, *GST*, *HSP70*, *UBQ*, *RPL32*, *Actin*, *UCCR*, *SDHA*, and *α*−*tubulin*. Among these genes, *SDHA*, *UCCR*, and *α*−*tubulin* exhibited the highest variation in expression, while the other 13 genes demonstrated lower variation (*EF1* > *RPS23* > *GST* > *UBQ* > *SOD* > *Actin* > *RPL32* > *AK* > *RPL7A* > *GAPDH* > *HSP70* > *RPS3* > *TATA*−*box*; Figure 1). Furthermore, the extent of variation in the expression of certain reference genes was influenced by experimental treatments. For instance, *α*−*tubulin* displayed lower variation (~1 cycle) for diverse tissues and adult age, but exhibited higher variation (>5 cycles) in other experimental treatments such as different developmental stages, temperature fluctuations, molting time treatment, and mating status (Figure 1).

### 3.3. Stability of Candidate Reference Genes

To determine the most suitable reference genes for six experimental conditions (development stages, adult tissues, adult age, nymph age, temperature, and mating status), the expression stabilities were assessed using ΔCt method, GeNorm, BestKeeper, and NormFinder. Subsequently, RefFinder was employed to compute an all-encompassing stability ranking.

#### 3.3.1. Developmental Stages

**Figure 2:**
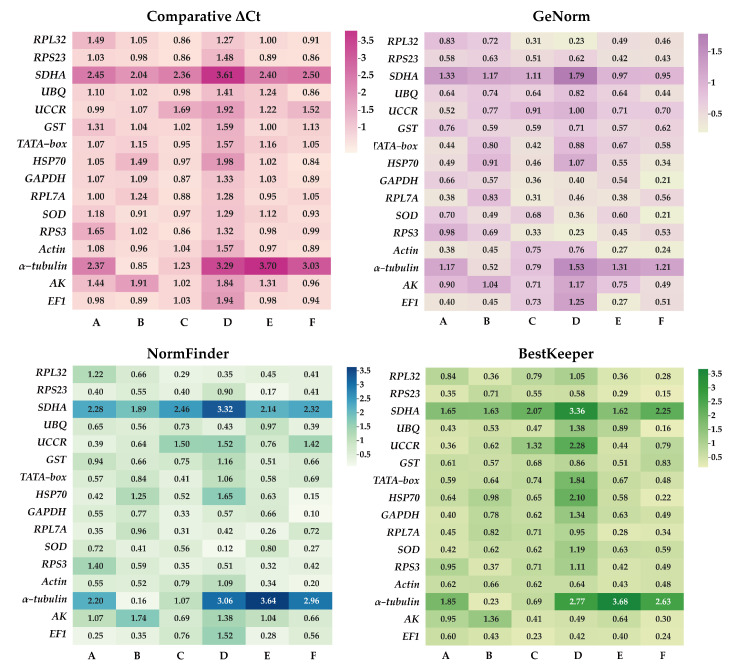
The stability of candidate reference genes assessed by NormFinder, BestKeeper, comparative ΔCt, and GeNorm. The candidate gene’s stability is indicated by the smaller value and lighter hues on the pane. A: Development stage, B: adult tissue, C: adult age, D: nymph age E: temperature, and F: mating status.

**Figure 3:**
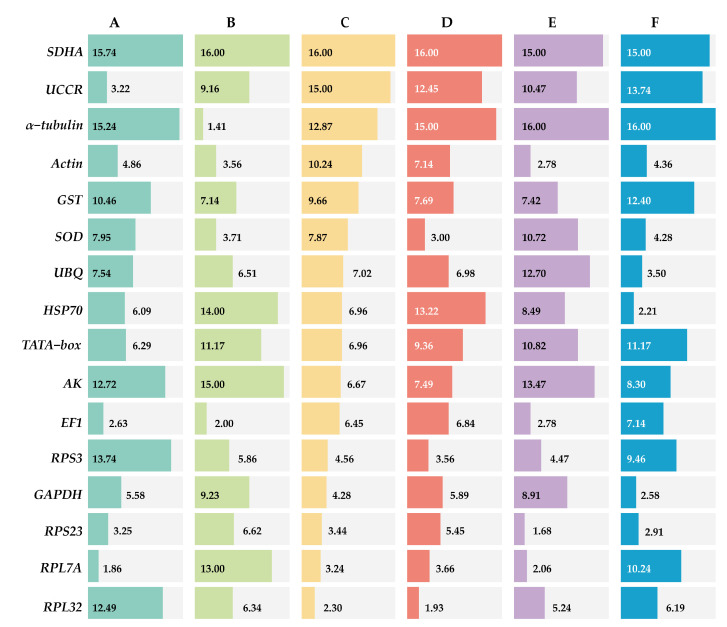
Expression stability of 16 candidate reference genes of *R. pedestris* in different treatments by RefFinder. A: Development stage, B: adult tissue, C: adult age, D: nymph age, E: temperature, and F: mating status.

**Figure 4:**
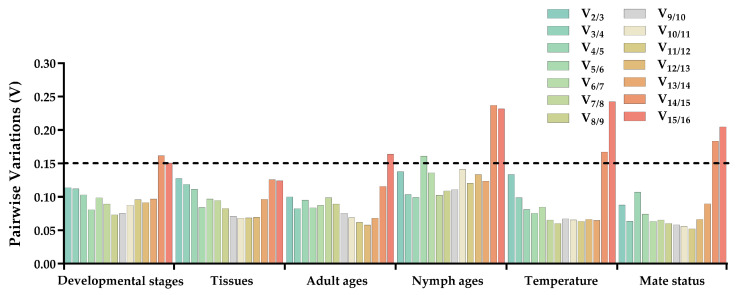
Optimal number of reference genes for normalization in qRT-PCR of *R. pedestris* under different conditions. GeNorm software was employed to analyze the pairwise variation (V_n/n+1_, where n represents the number of reference genes) in order to ascertain the appropriate number of reference genes necessary for precise normalization. The threshold for paired variation values (V_n/n+1_ = 0.15) is denoted by black dotted lines. A V_n/n+1_ < 0.15 signifies that the optimal number of reference genes for normalization is n, while a V_n/n+1_ > 0.15 indicates that the optimal number is n+1.

ΔCt method and GeNorm analysis revealed that *RPL7A* exhibited the highest stability across various developmental stages, while BestKeeper and NormFinder analyses identified *RPS23* and *EF1* as the most appropriate reference genes (Figure 2). Based on the RefFinder analysis, the expression stability was ranked as follows: *RPL7A* > *EF1* > *UCCR* > *RPS23* > *Actin* > *GAPDH* > *HSP70* > *TATA*−*box* > *UBQ* > *SOD* > *GST* > *RPL32* >*AK* > *RPS3* > *α*−*tubulin* > *SDHA* (Figure 3). According to the analysis using GeNorm software (Figure 4), the pairwise variation value V_2/3_ (0.113) did not exceed the predetermined threshold of 0.15, suggesting that the most suitable reference genes for normalization across developmental stages were *RPL7A* and *EF1* (Figure 3).

#### 3.3.2. Adult Tissues

According to ΔCt method and NormFinder analysis, *α*−*tubulin* and *EF1* were identified to be the most stable reference genes (Figure 2). Likewise, the GeNorm and BestKeeper programs indicated that *EF1* and *α*−*tubulin* were the most stable reference genes, respectively, while *SDHA* displayed the least stability (Figure 2). Based on the RefFinder program, the stability of reference genes was as follows: *α*−*tubulin* > *EF1* > *Actin* > *SOD* > *RPS3* > *RPL32* > *UBQ* > *RPS23* > *GST* > *UCCR* > *GAPDH* > *TATA*−*box* > *RPL7A* > *HSP70* >*AK* > *SDHA* (Figure 3). The analysis conducted with the GeNorm program revealed that the pairwise variation value V_2/3_ (0.127) fell below a threshold of 0.15 (Figure 4), indicating that both *α*−*tubulin* and *EF1* were suitable for normalization in the various adult tissues of *R. pedestris*.

#### 3.3.3. Adult Age

GeNorm and NormFinder analysis revealed that *RPL32* was the most stable gene for adult age. ΔCt method analysis, on the other hand, identified *RPL32* and *RPS23* as the most stable genes, while BestKeeper analysis preferred *EF1* and *AK* (Figure 2). RefFinder analysis further confirmed the stability ranking, with *UBQ* being the most stable gene followed by *RPL32* > *RPL7A* > *RPS23* > *GAPDH* > *RPS3* > *EF1* > *AK* > *TATA*−*box* > *HSP70* > *UBQ* > *SOD* > *GST* > *Actin* > *α*−*tubulin* >*UCCR* > *SDHA* (Figure 3). Additionally, GeNorm pairwise variation analysis indicated that *RPL32* and *RPL7A* were sufficient to normalize target genes in the context of adult age (Figure 4). Therefore, we recommend the combination of *RPL32* and *RPL7A* as the optimal reference genes for adult age.

#### 3.3.4. Nymph Age

For nymph age, GeNorm and NormFinder analysis revealed that the genes with the highest stability in expression were *RPL32* and *SOD*. ΔCt method analysis indicated that *RPL32* and *RPL7A* exhibited the greatest expression stability. According to BestKeeper analysis, *EF1* emerged as the most stable reference gene (Figure 2). Conversely, all four methods consistently demonstrated that *SDHA* had the lowest expression stability (Figure 2). The RefFinder program generated a comprehensive ranking of the expression stability of candidate reference genes, with RPL32 being the most stable, followed by *SOD*, *RPS3*, *RPL7A*, *RPS23*, *GAPDH*, *EF1*, *UBQ*, *Actin*, *AK*, *GST*, *TATA*−*box*, *UCCR*, *HSP70*, *α*−*tubulin*, and *SDHA* (Figure 3). Additionally, GeNorm analysis showed that the V_2/3_ value (0.09) was less than 0.15 (Figure 4), further supporting the selection of *RPL32* and *SOD* as the optimal combination of reference genes for nymph age.

#### 3.3.5. Temperature

*RPS23* was determined as the most stable gene for different temperature treatments according to ΔCt method and NormFinder. Additionally, *Actin* and *RPL7A* were identified as the most stable genes based on GeNorm. Finally, BestKeeper analysis revealed that *RPL7A* and *RPS23* were the optimal reference genes (Figure 2). The RefFinder ranking indicated that the stability followed the order *RPS23* > *RPL7A* > *Actin* > *EF1* > *RPS3* > *RPL32* > *GST* > *HSP70* > *GAPDH* > *UCCR* > *SOD* > *TATA*−*bo*x > *UBQ* > *AK* > *SDHA* > *α*−*tubulin* (Figure 3). Additionally, GeNorm pairwise variation analysis demonstrated that the normalization of target genes for different temperature treatments could be adequately achieved using two reference genes (Figure 4). Consequently, the optimal combination of reference genes for *R. pedestris* among temperature treatments was *RPL7A* and *RPS23*.

#### 3.3.6. Mating Status

*GAPDH* was identified as the most stable reference gene according to the NormFinder method and the GeNorm program (Figure 2). *RPS23* and *UBQ* emerged as the most stable reference genes when analyzed using BestKeeper software. Additionally, *HSP70* and *RPS23* were identified as the most stable genes based on ΔCt method (Figure 2). Furthermore, RefFinder program analysis revealed the stability ranking was as follows: *HSP70* > *GAPDH* > *RPS23* > *UBQ* > *SOD* > *Actin* > *RPL32* > *EF1* > *AK* > *RPS3* > *RPL7A* > *TATA*−*box* > *GST* > *UCCR* > *SDHA* > *α−tubulin* (Figure 3). Consequently, both *HSP70* and *UBQ* can be confidently selected and employed for normalization purposes in the mating status of *R. pedestris*, as indicated by the GeNorm and RefFinder programs.

### 3.4. Validation of Candidate Reference Genes in Different Conditions

**Figure 5:**
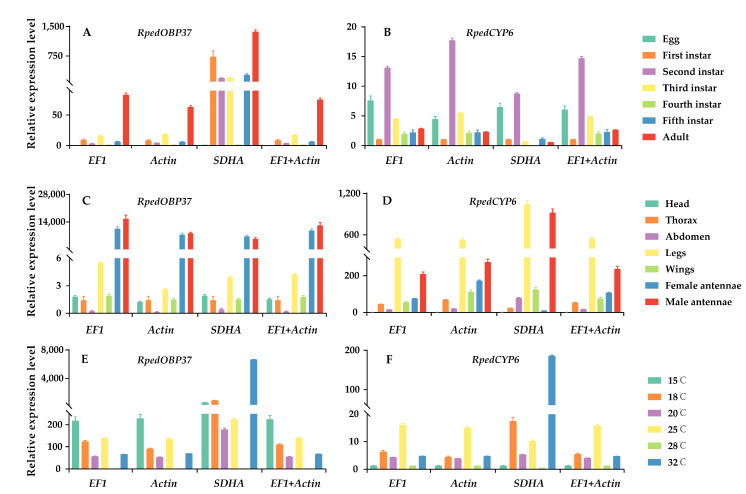
Expression level of *RpedOBP37* and *RpedCYP6* under different conditions in *R. pedestris*. The relative expression level of *RpedOBP37* and *RpedCYP6* were standardized using the most appropriate reference genes (*EF1* and *Actin*) and an unsuitable reference gene (*SDHA*). (**A**,**B**) *RpedOBP37* and *RpedCYP6* expression levels in developmental stages; (**C**,**D**) *RpedOBP37 and RpedCYP6* expression levels in various tissues; (**E**,**F**) *RpedOBP37* and *RpedCYP6* expression levels in different temperature conditions. The standard errors (±SE) were calculated from three biological replicates.

The target genes tested in this study were *RpedOBP37* and *RpedCYP6*, while three candidate genes (*EF1*, *Actin*, and *SDHA*) were utilized as reference genes to assess their expression level across different developmental stages, various tissues, and different temperature. When normalized with *EF1*, *Actin*, or a combination of *EF1* and *Actin*, *RpedOBP37* and *RpedCYP6* exhibited comparable expression patterns under different conditions (Figure 5). However, they showed more variations when gene expression was assessed by using the unstable gene, *SDHA*. Specifically, when *SDHA* was employed as the reference gene, a discernible disparity in the expression level of *RpedOBP37* was observed between the male and female antennae (Figure 5). Intriguingly, the male antennae exhibited a higher content of *RpedOBP37* compared to the female antennae, contradicting the findings for the other three groups.

## 4. Discussion

The quantification of qRT-PCR highly depends on the implementation of robust normalization techniques utilizing reference genes, which can mitigate the influence of variations in experimental data [33]. Nevertheless, an inappropriate reference gene potentially obscures or amplifies genuine biological alterations as a result of fluctuations in reference gene expression [34]. Additionally, relying solely on a single endogenous control can significantly impact the statistical outcomes and potentially result in erroneous data interpretation [35]. Numerous reports have focused on the evaluation of reference genes under diverse abiotic and biotic conditions in various insects, including *R. prolixus* [36], *Apolygus lucorum* [30], *Leptocybe invasa* [37], *Halyomorpha halys* [38], *Nezara viridula* [39], *Dichelops melacanthus* [40], and *Cnaphalocrocis medinalis* [13]. These studies have revealed that conventional reference genes generate substantial variations in the expression levels across different insect species and have no consistent expression patterns across all conditions. Consequently, the identification of suitable reference genes through careful screening becomes imperative for quantitative research conducted under specific conditions.

The genomic and transcriptomic data of *R. pedestris* have been published to study its development, chemosensory genes, and insecticide resistance [20,23,24,25,41,42]. Previous qRT-PCR studies on *R. pedestris* commonly employed universal reference genes for insect species [23,24,25,26]. Nevertheless, the use of inappropriate ones may result in misinterpretation of data. Hence, in this present study, we employed five software programs to evaluate the expression stability of 16 potential reference genes in *R. pedestris* across six distinct experimental conditions. The final evaluation results of RefFinder indicated that the selection of stably expressed reference genes exhibited variability across various developmental stages, adult tissues, adult ages, nymph ages, temperatures, and mating status.

There is no stable expression observed among reference genes, even the most commonly used ones, demonstrating variable expression levels are universal in different circumstances within the same insect species or across different insects under the same experimental conditions [9]. For instance, *Actin*, an extensively utilized reference gene, did not exhibit consistent expression stability in *R. pedestris* across all sample sets (Figure 3). The observation of significant variations in the expression levels of commonly used reference genes has been documented in multiple reports [10,29,30]. *SDHA*, which is commonly employed as an internal reference gene in animals, insects, and plants, exhibited inadequate expression stability in *R. pedestris* (Figure 2 and Figure 3). This notable disparity in expression stability has been consistently documented in numerous prior investigations [43,44]. *α−tubulin*, a traditional reference gene, exhibited significant expression stability exclusively in adult tissues, while displaying either low or the lowest expression stability in the remaining five experimental conditions. Comparable studies have been documented in investigating reference genes in Hemiptera, Coleoptera, and Lepidoptera species [10,29,45,46]. Previous research has shown that genes encoding ribosomal proteins consistently display stable expression and are widely used as reference genes in molecular studies of insects in the past decade [47]. The ribosomal protein family, including *RPS23* and *RPL32*, is also identified as the most suitable reference genes in insects [30,46] and other organisms [48,49,50], and it was ranked sixth by the ICG website (https://ngdc.cncb.ac.cn/icg/, accessed on 12 October 2023). In the present study, *RPS23* was assessed as the most stable gene in both nymph age and temperature treatments. Furthermore, *RPS23* demonstrated a relatively high level of stability in other treatments involving *R. pedestris*. Similarly, *RPL7A* and *RPL32* exhibited the highest degree of stability in both nymph age and temperature treatments, respectively (Figure 3). Additionally, these genes displayed a notable level of stability in other treatments. Prior studies have also demonstrated that ribosomal protein genes are commonly employed as reference genes across various Hemiptera insect species. For example, *RPL13A* and *RPS3A* are suitable for developmental stages and sexes of *Helopeltis theivora* [51]; *RPL27* and *RPL32* are suitable for different life stages and tissues of *A. lucorum* [30]; *RPL8* is suitable for various conditions of *C. hemipterus*, including developmental stage, adult tissue, adult sex, gas stimulation, and temperature [10]; *RPL29* was the most stable reference gene for various biotic conditions (host plant, acquisition of plant virus, developmental stage, and tissue) of *Bemisia tabaci* [52]. Nevertheless, ribosomal protein genes’ stability is not universally observed in all insect species [53].

The combination of multiple reference genes for normalizing gene expression in qRT-PCR analysis has garnered widespread acceptance due to its ability of yielding more precise and dependable expression patterns compared to a single gene [12,14]. Conversely, if the pairwise variation values exceed 0.15, we can select the three most stable reference genes as combinations based on the trend of the pairwise variation value [29,31]. In the current investigation, it was noted that the pairwise variation value for V_2/3_ under all conditions remained below the established threshold of 0.15 (Figure 4). As a result, it is advised to employ a combination of two internal references of *R. pedestris* for all treatment conditions. For example, *RPL7A* and *EF1* were optimal reference genes for different developmental stages, and *α−tubulin* and *EF1* were demonstrated as having the highest level of expression stability across diverse tissues. In the case of adult age, *RPL32* and *RPL7A* exhibited the most consistent expressions. *RPL32* and *SOD* displayed the most stable expression under various nymph age treatments. For temperature conditions, *RPS23* and *RPL7A* were identified as the most appropriate reference genes for monitoring gene expression. Finally, *HSP70* and *GAPDH* were recognized as the most dependable reference genes for assessing genes between mating status (Figure 3).

The practice of incorporating multiple reference genes to standardize the levels of target-gene expression has been widely accepted due to its ability to mitigate diverse errors and guarantee the precision of experimental outcomes [54,55]. Nevertheless, an inadequate or unreliable reference gene may result in an erroneous depiction of the target gene’s expression pattern, consequently leading to flawed interpretations [56,57]. The OBP gene plays a crucial role in the initial recognition processes of semiochemical perception and frequently exhibits expression patterns specific to certain tissues [58,59]. In the current study, notable variations in the expression level of *RpedOBP37* were observed across different tissues when normalized to the most stable reference genes (*EF1* and *Actin*) as well as an unstable reference gene (*SDHA*) (Figure 3). The detoxifying protein CYP6, which plays important roles in the response to oxidative stress in insects, demonstrates a wide distribution across various insect species [26]. When the expression levels of target genes were normalized using the most stable reference genes (*EF1* and/or *Actin*), *RpedCYP6* exhibited a consistent expression pattern. However, when the most unstable reference gene (*SDHA*) was utilized for normalization, the expression patterns of *RpedCYP6* did not align with those observed using the stable reference gene (Figure 5). Consequently, it is crucial to select and validate the most reliable reference genes for specific species under particular conditions.

## 5. Conclusions

In summary, this study employed five reputable algorithms to assess the stability of 16 candidate reference genes across diverse experimental conditions. The findings revealed that the combination of *RPL7A* and *EF1* exhibited the highest stability for developmental stages; *α−tubulin* and *EF1* for adult tissues; *RPL32* and *RPL7A* for adult age; *RPL32* and *SOD* for nymph age. Moreover, *RPS23* and *RPL7A* were the most stable reference genes under temperature treatments, while *HSP70* and *GAPDH* showed optimal stability for mating status. These results provide the suitable reference genes to conduct standardized qRT-PCR analysis on *R. pedestris*. This study offers a set of methodologies for the accurate normalization of qRT-PCR data, thereby establishing a fundamental basis for conducting functional investigations of target genes in *R. pedestris*.

## Figures and Tables

**Table 1:** Reference genes and specific primers used for qRT-PCR analysis in *R. pedestris*.

Gene	Accession No.	Primer Sequences (5′-3′, F/R)	Amplicon Length (bp)	Tm (°C)	Efficiency (%)	R^2^
*Actin*	Rped008782.1	AGTGGAGATGGCGTAACA	319	55	99.4	0.998
		GTGCTTCAGGTGCTTCAA				
*AK*	Rped016502.1	CCTTCCTTGTCTGGTGTAAT	386	55	96.6	0.998
		TCCGTCGTTCATCTCCTT				
*EF1*	Rped011851.1	TTGCCAACGGTTACACTC	135	55	104.4	0.995
		CGCCAGACTTGATAGACTTA				
*α*−*tubulin*	Rped009391.1	CCTCTGGCTATGCTCATC	292	55	101.9	0.999
		CGTTGCTCAGTTCCTCAT				
*RPL32*	Rped002411.1	GTTGTTGTACTGTATGAAGGAG	167	55	108.1	0.999
		TCAGGTGGCTTGATATTCTT				
*RPS23*	Rped002697.1	GGTATCAGAACTGCTAGGAA	389	55	113.0	0.994
		TTGTATAACGCCAGAAGAGA				
*SDHA*	Rped003372.1	GCTACTAGACTTCCAGGTATT	290	55	115.8	0.990
		TCACAGGCAATGGTCATAG				
*UBQ*	Rped005095.1	AACAGAGAACCAAGAATGC	270	55	108.2	0.995
		GAAGGACCCAAATGTAGAAC				
*UCCR*	Rped011417.1	TGATGGCAAGGCTAATGG	394	55	110.3	0.997
		TATGGAGGAAGGCTGGTT				
*GST*	Rped012521.1	AACCAGTTCGCCTAATGT	231	55	95.8	0.999
		CAACAGTTATATCCGCAAGA				
*TATA*−*box*	Rped001691.1	GAGGTTGAAGATGGTGGAA	369	55	109.5	0.999
		AGCCGCAAGAACTGAATT				
*HSP70*	Rped013173.1	TGTCCTCCTTGTTGATGTG	147	55	109.8	0.990
		GTTACTGCTGATTGGTTGTC				
*GAPDH*	Rped016319.1	GTTGTAGACCTCACTGTTAAG	240	55	113.5	0.992
		CGAATATCCGCACTCATTG				
*RPL7A*	Rped006873.1	CTAAGATCAAGCAGAAGGATG	155	55	108.2	0.999
		TGGCAGGAACAGAACAAG				
*SOD*	Rped004572.1	CAAGATGGTCCTACTTCTGA	283	55	96.2	0.999
		TCCTTCCAATGATGCTGAG				
*RPS3*	Rped018266.1	GTTGTGAAGTTGTTGTGTCT	202	55	102.9	0.997
		GGTCCATTCTTGCCAGTT				

## Data Availability

The data presented in this study are available on request from the corresponding author.

## Supplementary Materials

The following supporting information can be downloaded at: https://www.mdpi.com/article/10.3390/insects14120960/s1, Figure S1: Melting curve analysis of sixteen candidate reference genes; Figure S2: Standard curves of the sixteen candidate reference genes.
