# Supplementary material for: Evaluation of Reference Genes for Quantitative Real-Time PCR Analysis in the Bean Bug, Riptortus pedestris (Hemiptera: Alydidae)
Source: Insects. 2023 Dec 18;14(12):960. doi: 10.3390/insects14120960 (PMC10743553; doi:10.3390/insects14120960)
Supplement: Supplementary file 1 [file insects-14-00960-s001.zip › insects-2704274-supplementary.pdf]

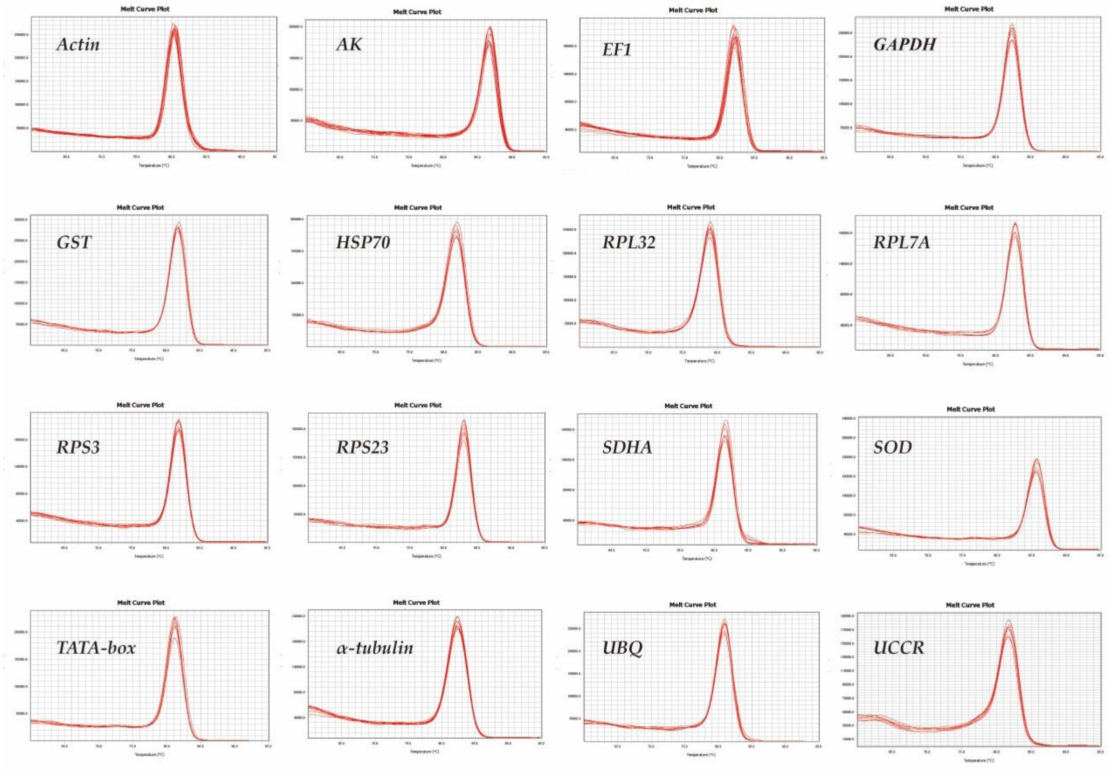

Figure S1. Melting curve analysis of twelve candidate reference genes.

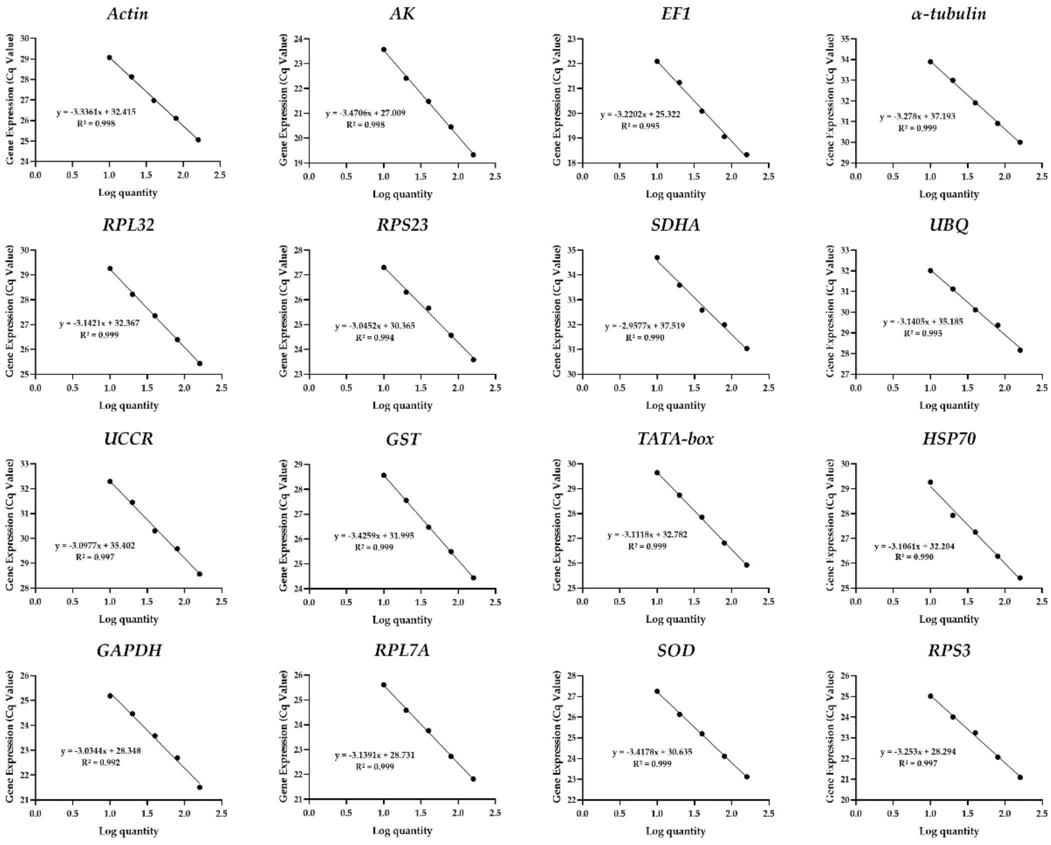

Figure S2. Standard curves of the twelve candidate reference genes.
